# Health effects of shrinking hyper-saline lakes: spatiotemporal modeling of the Lake Urmia drought on the local population, case study of the Shabestar County

**DOI:** 10.1038/s41598-023-28332-6

**Published:** 2023-01-28

**Authors:** Bakhtiar Feizizadeh, Tobia Lakes, Davoud Omarzadeh, Samira Pourmoradian

**Affiliations:** 1 Department of Remote Sensing and GIS, University of Tabriz, Tabriz, Iran; 2 Applied GIScience Lab, Humboldt-Universität zu Berlin, Berlin, Germany; 3 IRI THESys, Humboldt-Universität zu Berlin, Berlin, Germany; 4 IN3, Universitat Oberta de Catalunya, Barcelona, Spain; 5 Department of Community Nutrition, Faculty of Nutrition and Food Science, Nutrition Research Center, Tabriz University of Medical Sciences, Tabriz, Iran

**Keywords:** Environmental impact, Environmental sciences, Natural hazards

## Abstract

Climate change and its respective environmental impacts, such as dying lakes, is widely acknowledged. Studies on the impact of shrinking hyper-saline lakes suggest severe negative consequences for the health of the affected population. The primary aim was to investigate the relationship between changes in the water level of the hyper-saline Lake Urmia, along with the associated salt release, and the prevalence of hypertension and the general state of health of the local population in Shabestar County north of the lake. Moreover, we sought to map the vulnerability of the local population to the health risks associated with salt-dust scatter using multiple environmental and demographic characteristics. We applied a spatiotemporal analysis of the environmental parameters of Lake Urmia and the health of the local population. We analyzed health survey data from local health care centers and a national STEPS study in Shabestar County, Iran. We used a time-series of remote sensing images to monitor the trend of occurrence and extent of salt-dust storms between 2012 and 2020. To evaluate the impacts of lake drought on the health of the residences, we investigated the spatiotemporal correlation of the lake drought and the state of health of local residents. We applied a GIScience multiple decision analysis to identify areas affected by salt-dust particles and related these to the health status of the residents. According to our results, the lake drought has significantly contributed to the increasing cases of hypertension in local patients. The number of hypertensive patients has increased from 2.09% in 2012 to 19.5% in 2019 before decreasing slightly to 16.05% in 2020. Detailed results showed that adults, and particularly females, were affected most by the effects of the salt-dust scatter in the residential areas close to the lake. The results of this study provide critical insights into the environmental impacts of the Lake Urmia drought on the human health of the residents. Based on the results we suggest that detailed socioeconomic studies might be required for a comprehensive analysis of the human health issues in this area. Nonetheless, the proposed methods can be applied to monitor the environmental impacts of climate change on human health.

## Introduction

The significance of healthcare is undeniable. The prevalence of communicable and non-communicable diseases is rooted in various and sometimes invisible patterns of environmental phenomena. Some diseases, such as hypertension, can be caused by environmental phenomena such as the release of salt-dust scatter or air pollution^1^. Environmental risks have been shown to significantly impact human health, either directly, by exposing individuals to toxic agents, or indirectly, by affecting life-sustaining ecosystems^2^. According to the World Health Organization^3^, the environmental risk of air pollution is one of the leading universal public health issues and is responsible for about 9 million deaths per year. ​Environmental effects contribute to about 25% of diseases worldwide^4^. Environmental risks can take many forms. In recent decades, the environmental challenges associated with climate change, such as drought, deforestation, and desert extension, are increasingly widespread and severe and have significantly impacted the ecology of most of the lakes around the world.

Drought is one of the most tangible impacts of climate change. It leads to global environmental challenges such as water scarcity, decreased food production, and the drying of large lakes. Large lakes are significant freshwater sources that now face critical consequences due to climate change and associated phenomena^5^. Some noteworthy examples include Lake Urmia, Lake Chad, the Sea of Galilee, and Aral^6^. Salt-dust scattering occurs in areas where saline lakes are drying up and this can have severe negative impacts on the health of the residents in the area^7^. Salt-dust scatters are a type of atmospheric phenomenon linked to various environmental and climate impacts^8^. Dust scatters, which generally affect semi-arid and arid environments, are generated by thunderstorms or significant air pressure gradients that increase the wind speed across a large area^9^.

Salt-dust scatters have been occurring more frequently in temperate regions in recent years as a result of the effects of climate change, such as growing air pressure differences between the polar and tropic zones or prolonged drought periods^10,11^. From the centers of salt diffusion, salt-dust is scattered to the surrounding environment, including residential areas, causing significant air pollution. Particulate matter (PM), particles of variable but very small diameter, penetrate the respiratory system via inhalation, causing respiratory and cardiovascular diseases, reproductive and central nervous system dysfunctions, and cancer^1^. Therefore, the survey and monitoring of environmental phenomena that may endanger the health of the community should be prioritized.

In this context, earlier studies emphasized the determinative role of the environment on human health^4,7–9,12^, with many studies emphasizing the relationship between environmental changes and human health^3^. In a fragile ecosystem, such as that of a dying lake, the manifestation of various diseases can be expected^13,14^. These diseases can include tuberculosis, respiratory diseases, hypertension, asthma, eye diseases, pharynx and larynx diseases, kidney and liver diseases, hepatitis, and many more^13^. Exposure to air pollution causes various acute and severe health effects, such as respiratory and cardiovascular diseases^15^. As confirmed by earlier studies, air pollution and dusts scatters lead to tangible environmental issues associated with hypertension and a decline in general health of the affected population^1,16–19^. In addition, heavy metals, bacteria, viruses, and dangerous minerals may be carried by salt-dust scatters and inhaled by individuals, causing health problems^8^.

It is widely acknowledged that human health can be impacted by environmental factors, some of which can be catastrophic. Therefore, developing appropriate mitigation plans based on health vulnerability and risk assessments is critical to minimize the environmental impacts on human health^20^. In this context, vulnerability is an all-pervasive phenomenon in healthcare^21^. Geospatial health vulnerability mapping enables us to define and quantify vulnerability in geographic space and thus identify areas most in need of intervention^22^. Geographic Information System (GIS)-based spatial analysis allows us to model the spatial distribution of salt-dust scatters and their effects on human health. Due to the technological advancements over the past decade, the implementation of GIS spatial analysis for human health modeling and assessment has increased markedly. GIS spatial analysis aims to support the treatment of health problems in various geographic areas^4,7^. The ability of GIS to complement a range of different information sources associated with people and their locations also makes them particularly beneficial to human health specialists^23^. As GIS techniques support health planning, mapping of the environmental hazard, management of produced data, and spatial identification of human health vulnerability and risks, governments and organizations can effortlessly recognize the distribution and spread of disease across areas, and thus optimize the planning of intervention measures and monitor their effectiveness^24,25^.

Lake Urmia, one of the world’s largest dying lakes, has been facing the impacts of climate change for decades. Due to its hyper-salinity, it is expected to significantly impact its surrounding ecosystems. The salinity of the marginal lake, the increase in total salinity, the reduction of conventional agriculture, and salt-dust scatter are critical aspects of the dying lake^14^. Therefore, this research about the health of the local population in Shabestar County in relation to the dying lake Urmia has to main objectives: (1) To identify the relationship between changes in the water level of the hyper-saline Lake Urmia and associated salt release with the prevalence of hypertension in the local population. (2) To assess and map the risk of salt dust effects on human health.

## Study area

**Figure 1:**
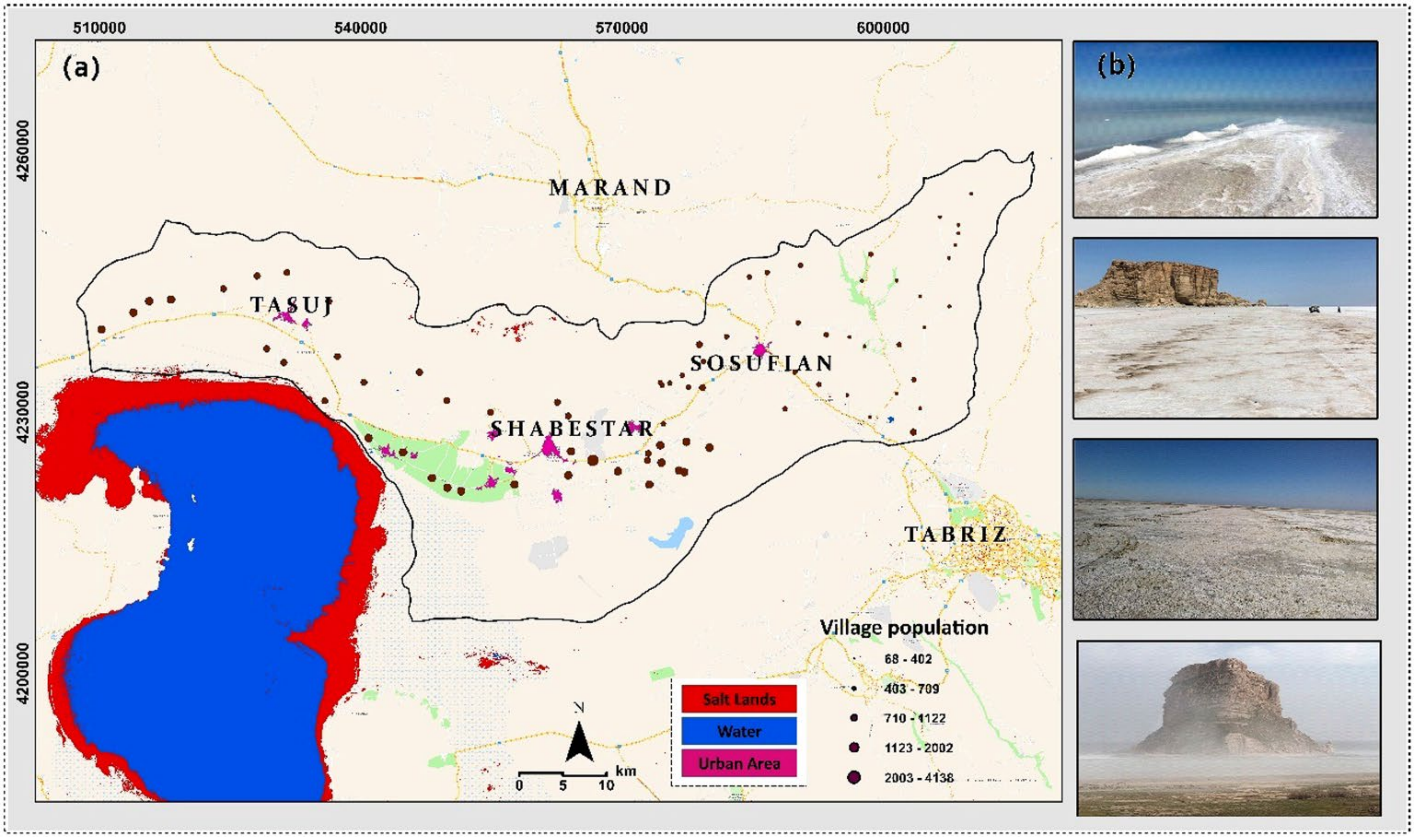
Location of the study area north of Lake Urmia (**a**), and several examples of salt-dust sources around the lake (**b**). The map is created in ESRI- Arc GIS version 10.7 under licenses of Humboldt-Universität zu Berlin (https://desktop.arcgis.com/).

**Figure 2:**
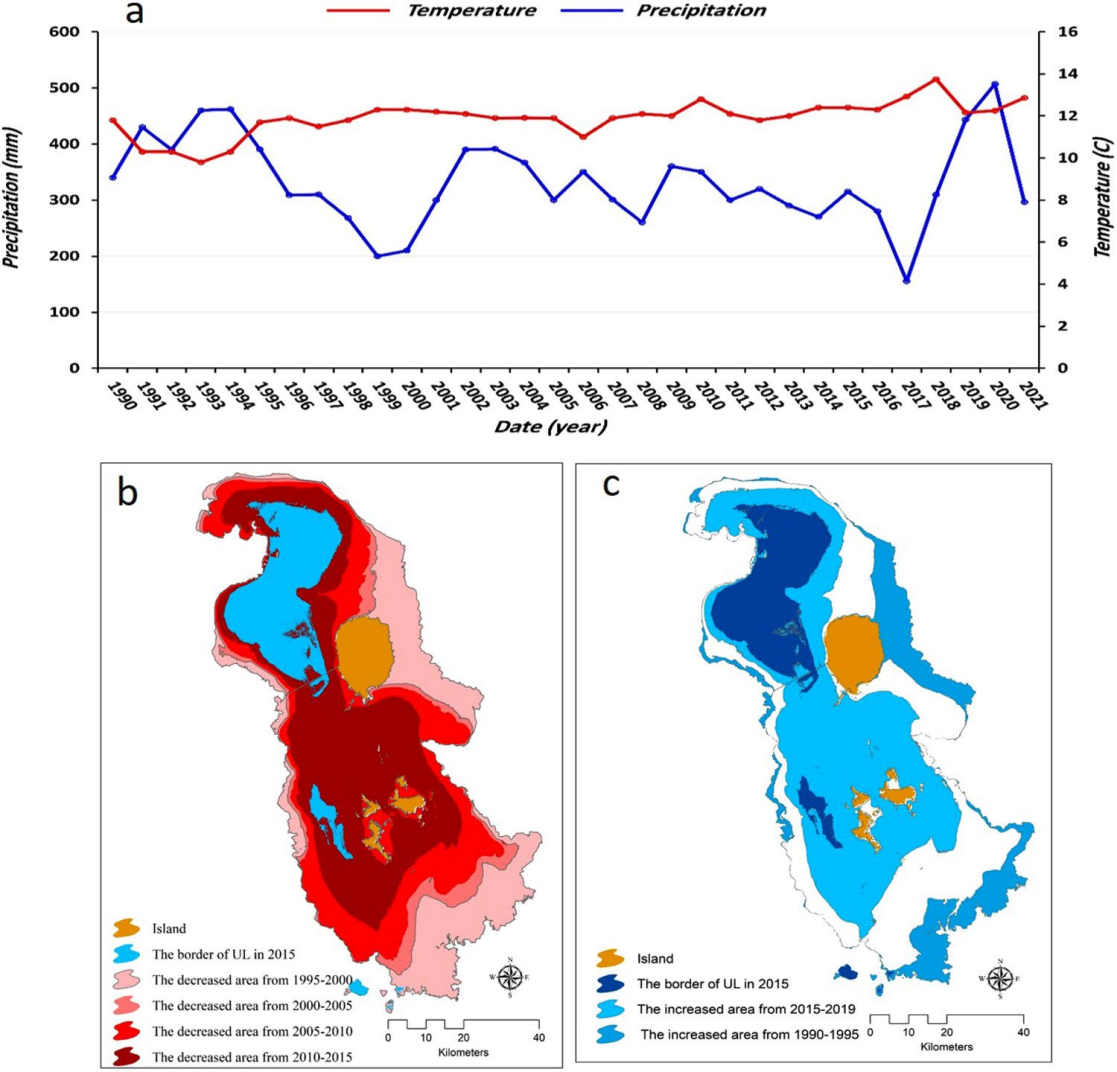
Trend of average annual precipitation and temperature, and the changes in surface area of Lake Urmia between 1990 and 2020. The map is created in ESRI- Arc GIS version 10.7 under licenses of Humboldt-Universität zu Berlin (https://desktop.arcgis.com/).

The study area, Shabestar County, with an area of 2631.45 km^2^, lies north of Lake Urmia in Iran. The county borders Mishow Mountain to the north and Lake Urmia to the south. The elevation increases from 1286 m at the lake bed to 3126 m in the Mishow mountain range (Fig. 1). The study area experience a semi-arid climate, with a maximum temperature about 40^○^C in summer is and minimum temperature of -18^○^ C in winter. The annual average rainfall is around 350 mm^26^. According to the 2016 census, the population of this county is 135,421 people. In recent years, changes in the water level of Lake Urmia have caused salt release centers in the southern and southwestern areas of Shabestar County. The Lake Urmia drought has led to dry soil, deforestation, an increase in airborne dust, and the dispersion of allergens, which potentially compromise respiratory and circulatory health^27^. Early studies for analyzing the lake drouth and its respective environmental issues pointed out the integrated impacts of intensive anthrophonic factors and climate change for lake drouth. In this regard, agricultural extension particularly with high water demand crops such as onion, tomato, water melon and etc., dam construction, and water diversions/withdrawals as reported as major anthrophonic factors contributed to the lake drought. The climate change impacts by means of increasing a temperature and drouth has also contributed over the past decades^26–30^. Figure 2a shows timeseries annual temperature and precipitation trends in lake Urmia basin. As this figure shows the annul precipitation has reduced since 1990 while the annual average temperature slightly increased. The impacts of anthrophonic factors and climate change for water level change and surface area of the lake is also represented in Fig. 2bc which clearly indicated an intensive exposed saline flow threating the ecosystems of area significantly.

Earlier studies also confirmed that drought leads to airborne salt-dust scatter^7,14,31–33^. When the saline flow sources around the lake are activated during monsoon season, they emit a wave of salt-dust into the air that can reach the neighboring cities^34^. The atmospheric aerosol is a complex and dynamic combination of solid and liquid particles affected by natural phenomena and human activities^35^. Due to the decrease in precipitation in recent years, the use of water for agriculture, construction of hydraulic structures and etc., surface area of the lake has been decreased. The predominant wind direction in Shabestar is westward. Considering the extensive exposed salt lands resulting from the lake drought and the direction of the wind, the coastal and even inland areas of Shabestar are in serious danger of salt intrusion^27^. There is a direct relationship between the scatter of salt particles in the air and the drying lake. The airborne particles are associated with serious illnesses^36^, as they can make their way into the airways and quickly reach the bloodstream. Aside from causing many diseases, salt release has also led to soil salinity, drying up the orchards in these areas (Fig. 1) and polluting the groundwater with heavy metals. According to some studies, the county's groundwater has reached contaminant concentrations exceeding WHO standards for drinking water^37–39^.

## Dataset and methods

### Data acquisition

We employed multi-temporal earth observation satellite imagery to detect and monitor the saline flow sources resulting from the Lake Urmia drought. The Spatial Data Infrastructure (SDI) project of the East Azerbaijan Province provided climatology, topography, and demography datasets of the study area at the scale of 1:25,000 (Table 1). To monitor the impacts of the drought on the public health of the study area, we consulted time-series health observation data from Shabestar Health Center and NIFHR^40^. We received several health indicators from the Health Center of Shabestar County, which we used to identify a trend of hypertension linked to the Lake Urmia drought. The details of data acquisition using the employed multi criteria approach are outlined in the following sections.

**Table 1:** Datasets employed in this study.

Main criteria	Sub-criteria	Sources
Demographic characteristics	Age group	Census in 2016
	Population density	
	Hypertension	STEPES survey and health care centers dataset from 2012 to 2020
	Land use/cover	Landsat Satellite images with spatial resolution of 30 m
	Vegetation continuous fields	Global Forest Cover Change-Surface Reflectance product, which is based on the enhanced Global Land Survey datasets
Geomorphological and land surface	Aspect	Topography dataset of the study area in the scale of 1–25,000
	Slope	
	Salt center	Landsat Satellite images
	Moisture	NASA-USDA SMAP Global soil moisture dataset
	Wind direction	Meteorology stations dataset and SDI project of the EAP
	Wind speed	

### Health data and hypertension status

To assess the lake drought and impacts of salt-dust scatters on the prevalence of hypertension in local residents, we employed time-series secondary health monitoring data from 2012 to 2020 obtained from the medical and health care centers in Shabestar County and the national STEPS project, which is funded by the Iranian Ministry of Health and Medical Education (I-MHME) under the ethics code IR.SBMU.RETECH.REC.1394.121^41^. The STEPS population-based survey of chronic disease risk factors considers Iranian adults aged 15–64, and those aged 25–64 for the biochemical measures^42^. In this survey, a cluster sample design was applied to produce representative data for the considered age range. A total of 12,000 adults participated voluntarily in the time-series survey^43^. This national study includes three phases, which are shown in Table 2 along with the data collected in each step. In step I, demographic data and behavioral measurements of all participants were collected through interviews. In the second step, the anthropometric indices, including height, weight, waist circumference, hip circumference, and systolic and diastolic blood pressure, were measured for all 12,000 participants. Finally, in the last phase, the biochemical measurements of the participant's ages between 25 and 64 were collected. This time series survey was carried out based on the international standards to reserve all human rights of the participants which are already acknowledged by I-MHME^41^. In order to support the relevant studies, the I-MHME makes these data available for researchers and scientists through certain policies such as research proposals, or official reasonable requests by universities and institutes. As the primary objective of our research was to investigate the relationship between changes in the water level of Lake Urmia and associated salt release and the prevalence of hypertension, we obtained the hypertension dataset collected through the STEPS project as secondary dataset to our study. In addition, in order to monitor the impact of the salt-dust scatters on the health of residents in the study area, we employed time-series health data obtained from 200 health care centers in cities and rural primary health care centers in rural areas. This dataset, which covers the timeframe of 2012 to 2020, includes the number of hypertension patients recorded in each health care center, their home location, and demographic characteristics. We also obtained the GPS location of the STEPS project participants’ area of residence. The survey included measuring the general health status of the 12,000 participants every three months as well as hypertension and other measures (see Table 2). To consider the living conditions and quality of life of the participants, they were also categorized based on their socioeconomic and lifestyle characteristics such as job, traveling plan on holidays, owned property (e.g., house, car, computer, etc.). In order to analyze the spatiotemporal patterns of the residents’ health, we mapped the spatial patterns using these datasets and GIS spatial analysis and interpolation techniques.

**Table 2:** Data gathered in each phase of the national STEPS project and the criteria observed in the survey analysis^41^.

Step I Physical activity	Tobacco use
	Alcohol consumption
	Fruit and vegetable consumption (in a typical week)
	Physical activity
Step II Physical measurements	Mean body mass index—BMI (kg/m^2^)
	Percentage who are overweight or obese (BMI ≥ 25 kg/m^2^)
	Percentage who are obese (BMI ≥ 30 kg/m^2^)
	Average waist circumference (cm)
	Mean systolic blood pressure—SBP (mmHg)
	Mean diastolic blood pressure—DBP (mmHg)
	Percentage with raised BP (SBP ≥ 140 and/or DBP ≥ 90 mmHg)
	Percentage with raised BP (SBP ≥ 160 and/or DBP ≥ 100 mmHg)
Step III Biochemical measurements	Mean fasting blood glucose (mmol/L)
	Percentage with raised blood glucose (≥ 7.0 mmol/L)
	Mean total blood cholesterol (mg/dL)
	Percentage total cholesterol (≥ 5.2 mmol/L)
	Percentage total cholesterol (≥ 6.5 mmol/L)

### Demographic characteristics

Demographic characteristics are one of the major aspects of the health and disease patterns within a society. It is well-known that the population characteristics such as age and gender, along with the anthropometric indices such as the lipid profile and the systolic and diastolic blood pressure, factor into the most common non-communicable diseases such as hypertension, type 2 diabetes, cancer, and chronic respiratory diseases, which affect the health-related quality of life of the susceptible population^44^ The STEPS projects already considered the demography of the 12,000 participants, but as we intended to provide the population density of the study area for the task of vulnerability and health risk mapping, we considered the detailed demographic information obtained using the latest census in 2016 for all 135,421 people in Shabestar County. We categorized the population into different classes based on the vulnerability to the salt-dust scatter hazard. Therefore, population data were classified from 15–25, 26–35, 36–45, 46–55, 56–65 based on the health STEPS survey dataset^45^. We also developed a population density map of the study area based on the census data.

### Geomorphological and land surface characteristics

We developed a digital elevation model (DEM) based on a 1:25,000 topographic map of the study area. We then used the DEM to generate slope and aspect maps. The slope and aspect were used as geographical indicators as they can affect the extent and distribution paths of salt and dust scatter^35^. Aspects facing the winds that blow from the lake in the east are more at risk, including western, southern, and southwestern slopes (180 to 360 degrees).

### Land use/cover and vegetation continuous percentage

Land use/cover can affect the spread of dust and salt particles around the lake. For instance, vegetated areas, especially with a dense tree cover, can prevent the spread of salt to residential areas, while bare lands or salt lands around the lake are the hot spots of the distribution of dust and salt particles. The land use/cover data for the year 2020 was obtained from our earlier studies regarding the Lake Urmia drought and its respective environmental impacts^46^. We also used the vegetation continuous fields (VCF) product to analyze the vegetation cover of the study area. The VCF represents the global surface coverage from 2000 to 2020, comprising data on tree cover, non-vegetative cover, and cloud cover. Given its global span and two-decade timeframe, the VCF has proven to be a valuable tool for researchers across disciplines. The VCF dataset is derived from the Global Forest Cover Change-Surface Reflectance product, which is based on the enhanced Global Land Survey (GLS) datasets. The GLS datasets are composed of high-resolution Landsat 5 and Landsat 7 images at a 30-m resolution from Google Earth Engine^47^.

### Saline sources

Since exposure to the saline sources is expected to significantly impact the residents’ health in the study area, we considered the distance to saline flows as one of the causal criteria. We obtained the saline diffusion center data from our earlier studies^47^. It is well understood that proximity to these saline diffusion centers both directly and indirectly affects the prevalence of respiratory diseases and hypertension. We thus considered the areas within 80 km of saline diffusion sources.

### Climate criteria

The spatial distribution of salt-dust scatters is closely correlated with the climate characteristics. The following criteria were considered to represent the climate characteristics of the study area.

### Soil moisture

Soil moisture plays an important role in releasing salt particles and dust into the air. Particles are transported by the wind more easily in areas where the soil moisture is low. We, therefore, employed the NASA-USDA Global Soil Moisture Dataset to represent the soil moisture of the study area. The NASA-USDA Global soil moisture and the NASA-USDA SMAP Global soil moisture dataset provide soil moisture information across the globe at a 0.25° × 0.25° spatial resolution. Values around 0 indicate typical moisture conditions, while very positive and very negative values indicate extreme moisture (soil moisture conditions above average) and dryness (soil moisture conditions below average), respectively^48^.

### Wind speed

Wind speed is another main factor affecting the impact of salt-dust on the health of the residents. Thus, the wind speed data of meteorology stations were collected from the SDI project of the EAP, and wind speed and direction maps were obtained based on interpolation techniques. Based on this dataset, we consider winds speed of 14–17 knots (7–8.5 m/s) to be causal indicators in the study area.

### Wind direction

The wind direction determines the distribution of dust and salt particles in the areas around Lake Urmia. The wind ordinarily blows from north to south and from east to west in the study area. In some cases, in the north of Lake Urmia, the wind direction changes from west to east and affects the study area.

## Methodology

**Figure 3:**
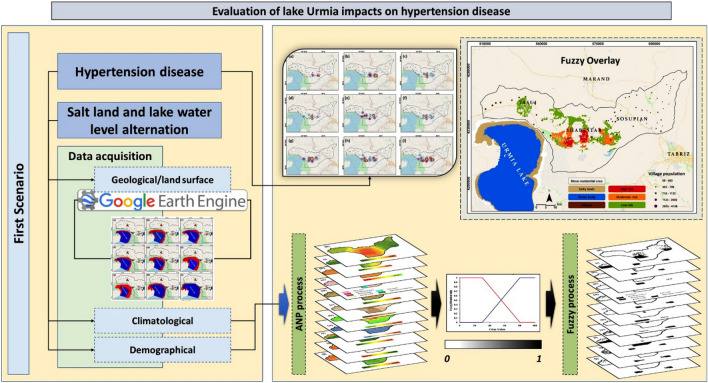
Main steps of the implemented research methodology for human health risk mapping using integrated spatiotemporal techniques.

The research methodology was established based on GIScience's geospatial analysis and spatiotemporal modeling techniques. Landsat earth observation satellite images were used to obtain the trend of exposed salt lands in the area north of Lake Urmia. For this goal, we employed timeseries Landsat satellite images from July 2012 to 2020 provided by NASA/USGS. All image processing performed in geospatial platform of Google Earth Engine. In the second step, a GIS spatial analysis was applied to map the hotspots of hypertension using Moran's I Index. In addition, a GIS-based multi-criteria decision analysis (GIS-MCDA) approach was also used to develop a public health risk susceptibility map based on the relevant indicators. Figure 3 gives an overview of the research methodology.

### Trend analysis of the of salt-dust scatter sources

Our first step was to investigate the relationship between changes in the water level of Lake Urmia and associated salt release and the prevalence of hypertension. Therefore, we applied remote sensing time-series satellite imagery to monitor the multi-temporal trend of lake changes. An integrated deep learning and machine learning data-driven algorithm was applied to determine the saline flow sources and salt-dust scatter sources around the lake. We refer to our earlier work in his area for the detailed approach, results, and their validation^7^.

### Spatiotemporal hypertension hotspot analysis

GIS-based spatial analysis provides unique capabilities for hotspot mapping. Since the survey data was recorded in the GPS points format, we were able to apply a GIS-based point pattern analysis to determine the spatiotemporal pattern of hypertension. Therefore, in the first step, the Kernel Density Estimation technique was applied to obtain the spatial pattern of the hypertension dataset. In the second step, the Global Moran’s Index (GMI) was applied to detect the hypertension hotspots in the study area. The GMI is one of the most efficient spatial autocorrelation assessment methods in GIS^49,50^. The GMI is frequently used to depict a spatial relationship based on spatial attributes, which can be used to analyze the location of impacting criteria such as land characteristics. We employed the GMI method to investigate the spatial correlation of the hypertension hotspots with environmental indicators (e.g., land characteristics, climate, etc.). The geographical distribution highlights a complicated distribution and spatial patterns. Thus, the GIS-based GMI can be used to investigate the spatial correlation to determine whether nearby areas have similar or dissimilar values^51^. The spatial correlation measures the spatial dependency between the values of random variables in different geographical regions^50,52^. The GMI spatial correlation index is calculated using Eq. (1):

1 $$I = \frac{{n\Sigma \Sigma w_{ij} (x_{i} - \overline{x})(x_{j} - \overline{x})}}{{w\Sigma (x_{i} - \overline{x})^{2} }}$$

where *Xi* is the relative distance correlation between area units of *i*, *N* is the number of area units, and *Wij* is the weight. The GMI value ranges from 1 to − 1. A number close to 1 indicates grouped patterns in areas with similar values (high or low), whereas a value around − 1 indicates dispersed patterns. Random patterns are indicated by a value close to zero^53^.

### GIS-MCDA for HHR mapping

**Figure 4:**
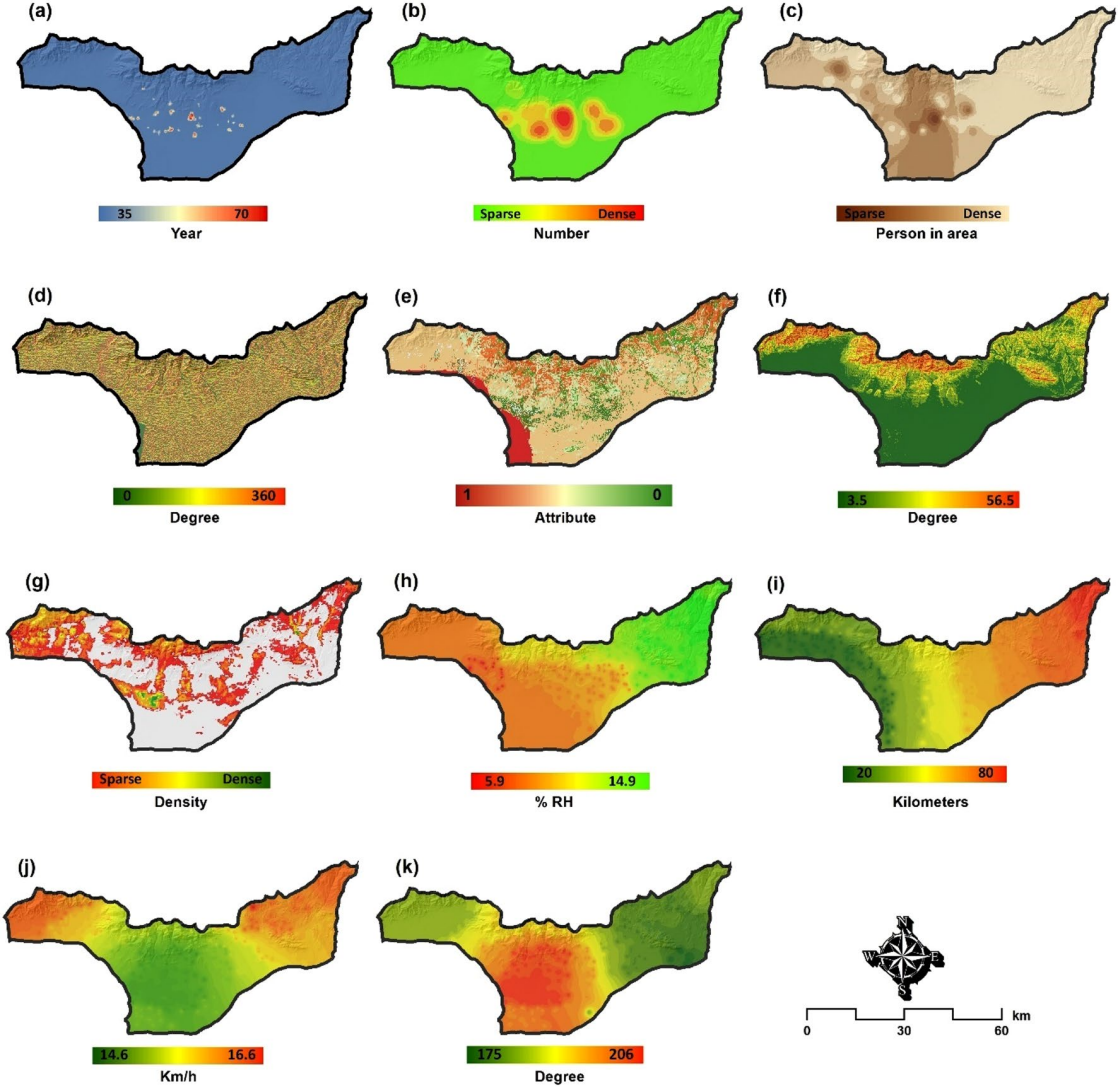
Spatial distribution of the selected criteria for the vulnerability and health risk mapping criteria: (**a**) population age group, (**b**) current status of hypertension, (**c**) population density, (**d**) slope-aspect, (**e**) land use/cover, (**f**) slope, (**g**) vegetation continuous fields, (**h**) moisture, (**i**) distance to salt-center, (**j**) wind speed and (**k**) wind direction. *Note*: these data are taken from the initial source (e.g., topography, climatology, demography, or health dataset used in this research). The map is created in ESRI- Arc GIS version 10.7 under licenses of Humboldt-Universität zu Berlin (https://desktop.arcgis.com/).

GIS-based Multi-Criteria Decision Analysis (GIS-MCDA) methods have become prevalent in environmental modeling and natural hazard prediction^54^. Of the spatial decision-making methods, the MCDA produces the most consistent judgement as it has a better structure and methods for obtaining and translating human judgements into priorities^55^. GIS-MCDA methods support meaningful spatial decisions by integrating multiple criteria from various spatial data sources^54,56,57^. Leveraging this capability, we used 11 criteria in three major groups to analyze and develop human vulnerability and health risk maps. The selection of the relevant criteria was based on the data availability and research literature^23,58–62^. Table 3 shows the list of selected criteria for vulnerability and health risk mapping. After identifying the relevant criteria, the required geometric and topological editing was carried out to develop the criteria into a 20 m-resolution spatial dataset and stored in a geodatabase for further analysis (Fig. 4).

**Table 3:**
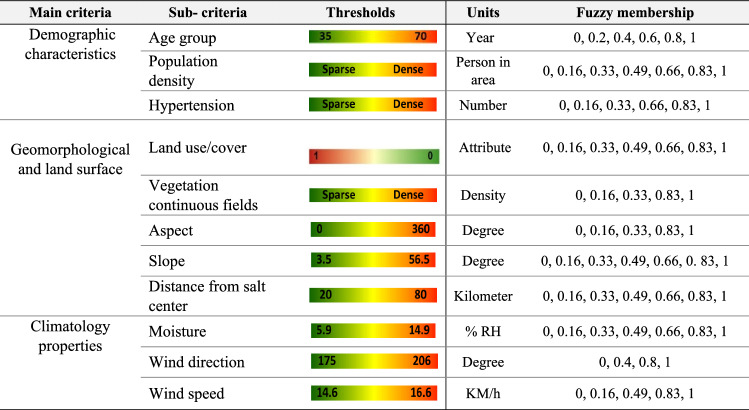
The internal classes of each criterion and their fuzzy memberships which are standardized at a scale of 0–1 based on the significance of each criterion for the vulnerability and health risk.

**Figure 5:**
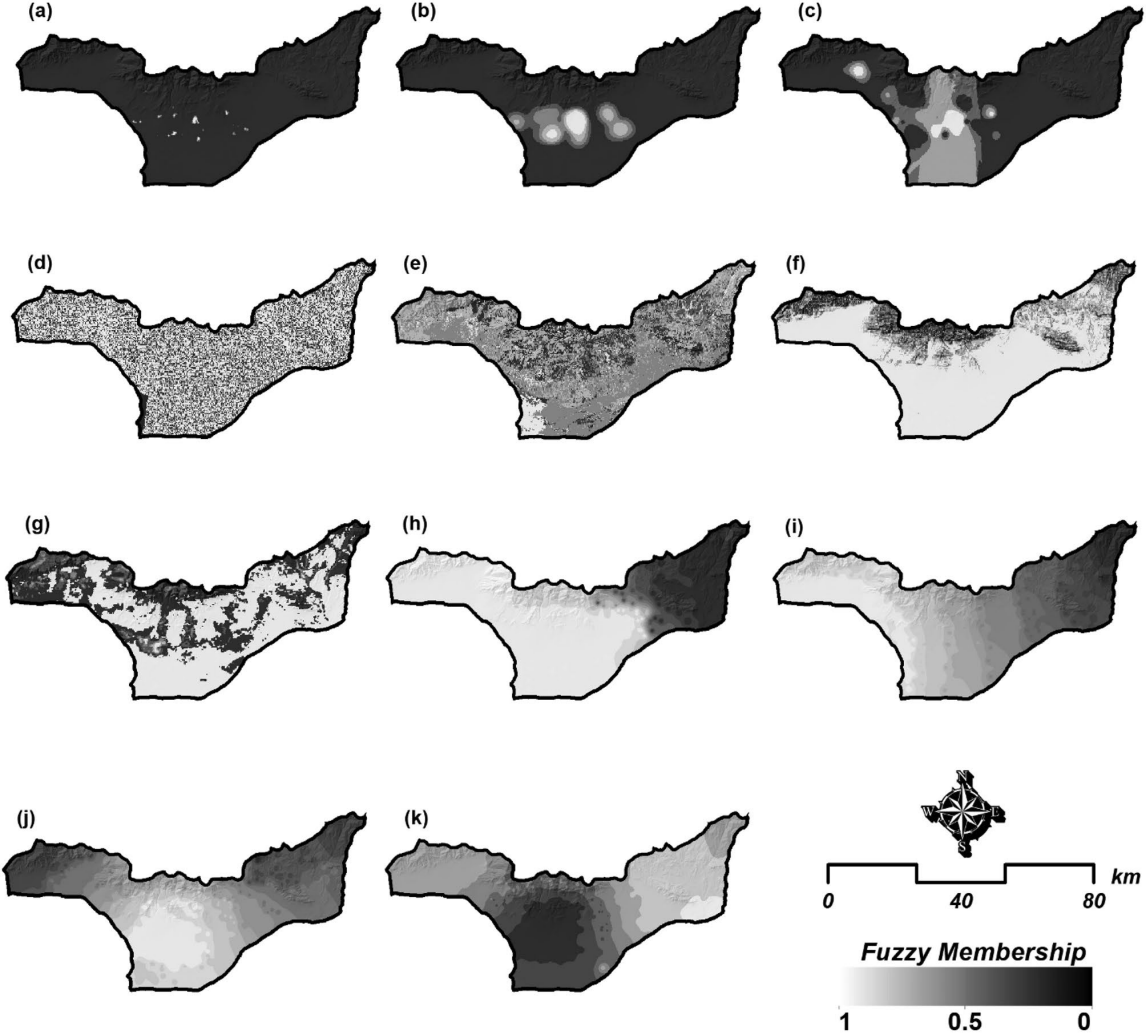
Fuzzy-based standardized layers of the selected criteria shown in Fig. 4 for the vulnerability and health risk mapping: (**a**) age group, (**b**) current status of hypertension, (**c**) population density, (**d**) slope-aspect, (**e**) land use/cover, (**f**) slope, (**g**) vegetation continuous fields, (**h**) moisture, (**i**) distance to salt-center, (**j**) wind speed, and (**k**) wind direction. *Note*: due to heterogeneous contexts of the indictors, we standardized all criteria in fuzzy scale for the spatial aggregation of HH-VRM. The map is created in ESRI- Arc GIS version 10.7 under licenses of Humboldt-Universität zu Berlin (https://desktop.arcgis.com/).

In GIS-MCDA, standardization is critical due to the variety of data sources and the different measurement scales. To facilitate standardization, fuzzy logic can be employed as a mathematical basis for incorporating different variables and decision systems with varying levels of ambiguity^56^. As Table 3 shows, the selected criteria have different values (e.g., degree, percent, kilometer, etc.) depending on the nature of the initial data source it was prepared from (e.g., topography, climatology, demography, or health dataset). In the GIS-MCDA process, all selected criteria can either be considered as beneficial criteria, where a higher score is favorable (e.g., wind speed), or cost criteria, where smaller scores are favorable (e.g., distance to salt centers). Thus, in order to equalize all criteria, a standardization was applied using the fuzzy method considering the cost -benefit context at a scale of 0–1. In this standardization, pixels with a value of 1 have complete membership, and pixels with a value of 0 have no membership. Therefore, after the standardization step, all criteria were converted to values between 0 and 1 based on the linear function. Figure 5 represents the standardized spatial distribution of the selected criteria affecting the onset and increase of respiratory diseases. Table 3 also shows the initial classes of each criterion and their fuzzy memberships.

### Criteria weighting and sensitivity analysis

GIS-MCDA-based spatial modeling requires the significance of each criterion to be determined. Criteria weighting must thus be applied to establish the importance of each criterion^54^. We employed the integrated fuzzy analytical network process (FANP) approach for criterion weighting. FANP is one of the most widely used methods for calculating criteria weights^63–66^, and previous studies attest to its effectiveness^7,56,57^. The integrated FANP technique can be used to calculate the intrinsic weight of each criterion. We applied the FANP to the initial criteria ranking obtained in the pairwise comparison matrix based on the expert knowledge of 30 specialists from the department of environmental health at the Medicine University of Tabriz, who ranked the criteria from 1 to 9 based on the significance of each criterion for vulnerability and health risk. Then, the pairwise comparison matrix was developed using the *Super Decisions* software, and the criteria weights were computed (Table 5).

**Figure 6:**
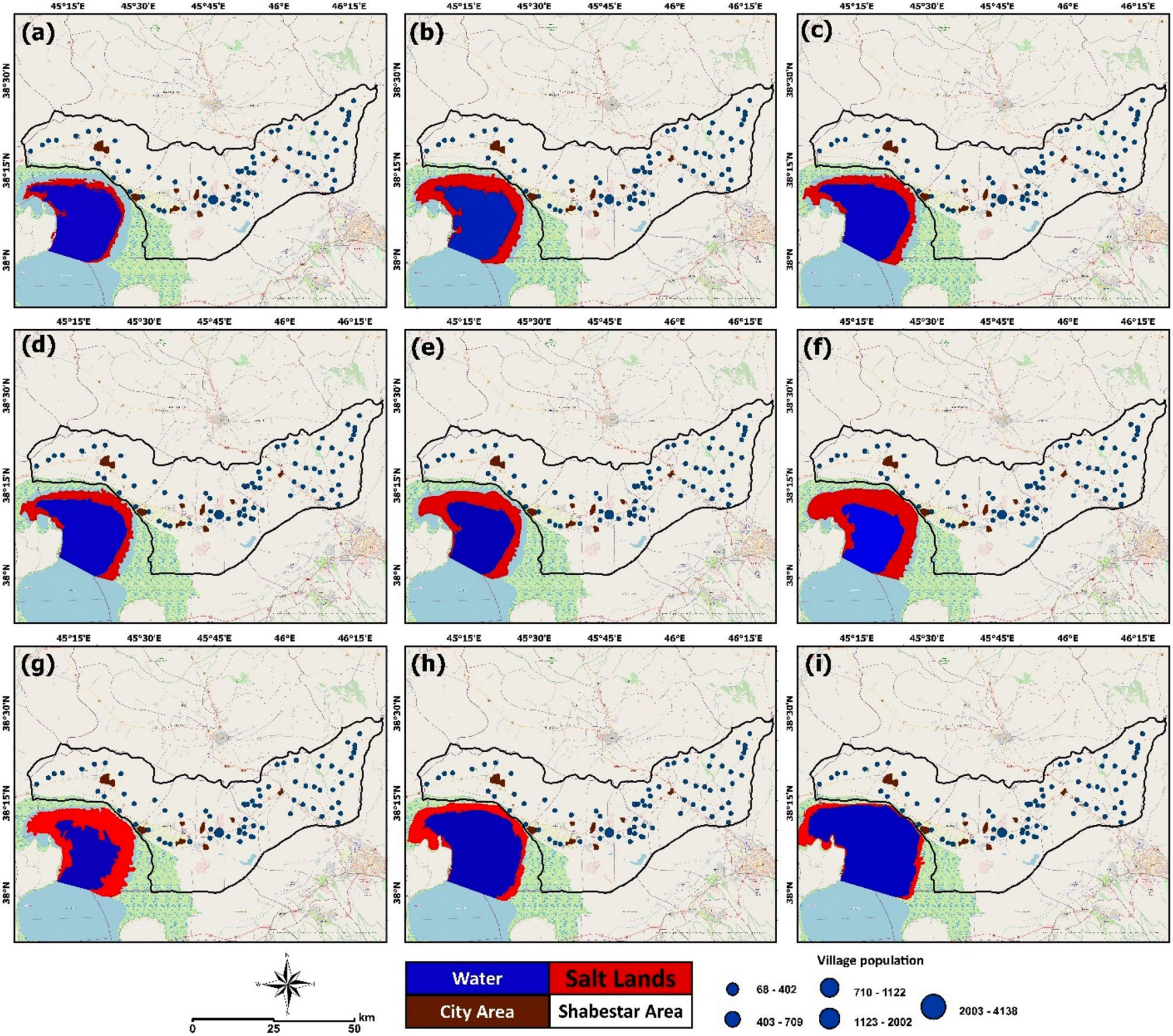
Results of Lake Urmia drought monitoring and the extension of salt-dust scatter sources around the lake in: (**a**) 2012, (**b**) 2013, (**c**) 2014, (**d**) 2015, (**e**) 2016, (**f**) 2017, (**g**) 2018, (**h**) 2019, and (**i**) 2020. These maps are based on the time-series satellite images and remote sensing change detection analysis. The map is created in ESRI- Arc GIS version 10.7 under licenses of Humboldt-Universität zu Berlin (https://desktop.arcgis.com/).

To standardize the layers, we employed the fuzzy membership function in GIS. The linear function was applied to transform the categorized raster layers into fuzzy standard layers with pixel values ranging from 0 to 1. The FANP method combines the analytical network process method with the fuzzy level evaluation method^67^. Furthermore, the fuzzification allows us to objectively analyze qualitative factors in complex decision-making using fuzzy mathematics membership theory^68^. 11 standardized raster maps were obtained, each with values ranging from 0 to 1. The regional distribution of the selected and standardized criteria is depicted in Fig. 6. The impact of each layer class on HHR was used to establish superiority and value allocation.

Due to the various data sources, expert knowledge for criterion ranking, and modeling faults, uncertainty in GIS-MCDA is inevitable^69^. Criteria weighting contributes significantly to the ambiguity of the MCDA framework in this case. According to earlier studies, such an ambiguity can potentially lead to incorrect results^70^. Thus, earlier studies proposed a sensitivity analysis as a means to overcome this issue^47,71^. In this study, the sensitivity analysis of the criteria weights produced from the FANP was computed using an integrated sensitivity analysis. Therefore, we applied a sensitivity analysis to examine the uncertainty associated with the FANP's weights for spatial modeling to reduce the risk of mistakes in our GIS-MCDA-based decision model. Our integrated approach was established based on the Monte Carlo simulation (MCS) and a global sensitivity analysis (GSA) to calculate the uncertainty of the criteria weights. The MCS was computed as follows:

2 $$\mathrm{MCS}:\mathrm{ T}=\sqrt{\frac{1}{\mathbf{n}}{\sum \left(\begin{array}{c}\frac{{{\varvec{\updelta}}}_{\mathbf{i}}}{{\mathbf{w}}_{\mathbf{i}}}\end{array}\right)}^{2}}$$

where *T* is the simulation, *w*_*i*_ is the random number and *N* indicates the simulation number. The GSA can also be computed using the following equations:

3 $${S}_{i}={v}_{i}/v$$

Equation (3) is known as ‘interactions’. A model without interactions among its input factors is considered as additive. In this case, $\sum_{i=1}^{k}S=1$, and the first-order conditional variances are necessary in order to decompose the model output variance. For a non-additive model, higher order sensitivity indices, which are responsible for interaction effects among sets of input factors, must be computed. However, higher order sensitivity indices are usually not estimated, as in a model with *k* factors the total number of indices (including the *S*_*i*_*s*) that should be estimated is as high as 2^k^ − 1. For this reason, a more compact sensitivity measure is used. This is the total effect sensitivity index, which concentrates all the interactions involving a given factor *X*_*i*_ in one single term. For example, for a model of *k* = 3 independent factors, the three total sensitivity indices would be as follows^73^.

4 $${S}_{T1}=\frac{V\left(Y\right)- {V}_{{X}_{2}{X}_{3}}\left\{{E}_{{X}_{1}}(Y|{X}_{2},{X}_{3})\right\}}{\mathrm{V}(\mathrm{Y})}={S}_{1}+{S}_{12}+{S}_{13}+{S}_{123 }$$

Analogously:

5 $${S}_{T2}={S}_{2}+{S}_{12}+{{S}_{23}+S}_{\begin{array}{c}123, \\ \end{array}}$$

6 $${S}_{T3}={S}_{3}+{{S}_{13}+{S}_{23}+S}_{\begin{array}{c}123. \\ \\ \\ \end{array}}$$

In other words, the conditional variance in Eq. (8) can be generally written as ${V}_{X-i\left\{{E}_{X-i}(Y|X-i)\right\}}$^73^. It expresses the total contribution to the variance of *Y* due to non-*X*_*i*_, i.e. to the *k* − 1 remaining factors, hence $V\left(Y\right)-{V}_{X-i}\left\{{E}_{X-i}(Y|X-i)\right\}$ includes all terms, i.e. a first-order term as well as interactions in Eq. (4), which involve factor *X*_*i*_. In general, $\sum_{i=1}^{k}{S}_{Ti}\ge 1$, with equality if there are no interactions. For a given factor *X*_*i*_ a notable difference between *S*_*Ti*_ and *S*_*i*_ flags an important role of interactions for that factor in *Y*. Highlighting interactions between input factors helps us to improve our understanding of the model structure^73^. In the context of implementation variance-based GSA we continued the analysis from calculating the importance of spatial bias in determining option rank order by means of Average Shift in Ranks (ASR) as follows^69^:

7 $$ASR = \frac{1}{n}\sum\limits_{{a = 1}}^{n} {\left| {a\_rank_{{ref}} - a\_rank} \right|}$$

where ASR is the average shift in ranks, *a_rank*_*ref*_ is the rank of option *A* in the reference ranking (e.g. equal weight case), and *a_rank* is the current rank of option *A*. ASR captures the relative shift in the position of the entire set of options and quantifies it as the sum of absolute differences between the current option rank (*a_rank*) and the reference rank (*a_rank*_*ref*_), divided by the number of all options^69^. In this regard, the original FANP weights can be applied as input weights for MCS and GSA-based sensitivity analysis. Thus, the MCS-GSA implementation consists of four essential steps, namely: (a) obtaining training data from the results of the hypertension hotspot analysis, as discussed above, (b) using the FANP's weights as reference weights, (c) running the MCS-based simulation 10,000 times, and (d) computing the spatial distribution of ranks (minimum, maximum, average, and standard deviation) results using the inverse weighted distance spatial interpolation method. As part of the GSA implementation, we also computed the two critical indices of *S* (first-order) and *St* (second-order) using the GSA approach (total effect). Technically, the *S* and *St* stand for the FANP's weights, which are indicated semantically. The uncertainty associated with the criteria weights can be defined as any difference between the value and order of the *S* and *St* indexes and the reference weights (e.g., FANP's weights). A considerable disparity in the values of *S* and *St* suggests that the reference weights (e.g., FANP) are questionable, which could lead to erroneous conclusions. Table 4 illustrates the evaluated criteria, the FANP weights that were obtained as reference weights, and the GSA sensitivity analysis results. The difference between *S* and *St* is not significant, indicating that the computed FANP weights can be used in data aggregation.

**Table 4:** Results of criteria weighting using the Fuzzy Analytical Process and sensitivity analysis based on the Monte Carlo and Global Sensitivity Analysis to validate the obtained indicator weights for vulnerability and health risk mapping.

Main criteria	Sub-criteria	FANP’s weights	*S*	*St*
Demographic characteristics	Age group	0.12	0.051	0.054
	Population density	0.048	0.061	0.068
	Hypertension	0.34	0.157	0.164
	Land use/cover	0.04	0.013	0.018
	Vegetation continuous fields	0.025	0.042	0.028
Geomorphological and land surface characteristics	Aspect	0.015	0.009	0.012
	Slope	0.015	0.083	0.046
Climatological characteristics	Salt Center	0.177	0.182	0.198
	Moisture	0.055	0.152	0.148
	Wind direction	0.151	0.132	0.141
	Wind speed	0.0038	0.006	0.009

### Spatial aggregation and validation

The final maps were created using the computed weights and geographical data aggregation of the required criteria. The ordered weighted average method (OWA) is a popular spatial aggregation method in GIS that has been supported by research [e.g.,^57^]. In this numerical technique, the geographical criteria from one map layer are integrated with the attributes (numerical) of the other criteria^72^. The OWA is one of the most efficient data aggregation models in GIS that uses fuzzy decision rules for data aggregation and provides a parameterized class of MCDA aggregating operators. OWA provides a parameterized class of multi-criteria aggregation operators between the minimum and the maximum. For a given set of *n* criterion (attribute) aps, an OWA operator can be defined as the following function $\underset{}{\mathrm{OWA}:{\mathrm{I}}^{\mathrm{n }}\to \mathrm{I}}$, where I = [0, 1] that is associated with a set of order weights V = [${\mathrm{v}}_{1, }{\mathrm{v}}_{2 , }\dots , {\mathrm{v}}_{\mathrm{n}}$] so that ${\mathrm{v}}_{\mathrm{j }}\upepsilon [\mathrm{0,1}]$ for j = 1, 2,…, n and ${\sum }_{\mathrm{j}=1}^{\mathrm{n}}=1{\mathrm{v}}^{\mathrm{j }}=1$ given a set of standardized criterion value ${\mathrm{A}}_{\mathrm{i }}=[{\mathrm{a}}_{\mathrm{i}1 }, {\mathrm{a}}_{\mathrm{i}2 }, \dots , {\mathrm{a}}_{\mathrm{in }}]$ for i = 1,2,…m, where ${\mathrm{a}}_{\mathrm{ij }}\upepsilon [\mathrm{0,1}]$ is associated with the location (e.g., cell, polygon, line, point), the OWA operator is defined as follows:

8 $${\text{OWA}}_{{\text{i}}} = [{\text{a}}_{{{\text{i}}1}} ,{\text{a}}_{{{\text{i}}2}} , \ldots ,{\text{a}}_{{{\text{in}}}} ] = \sum\limits_{{{\text{j}} = 1}}^{{\text{n}}} {{\text{v}}_{{\text{j}}} {\text{z}}_{{{\text{ij}}}} }$$

where ${\mathrm{za}}_{\mathrm{i}1 }\ge {\mathrm{z}}_{\mathrm{i}2 }\ge \dots \ge {\mathrm{z}}_{\mathrm{in}}$ is the sequence obtained by reordering the criterion values ${\mathrm{a}}_{\mathrm{i}1 }, {\mathrm{a}}_{\mathrm{i}2 }, \dots , {\mathrm{a}}_{\mathrm{in}}$. With different sets of order weights $\mathrm{V}$, one can generate a wide range of OWA operators by changing the set of order weights $\mathrm{V}$^46^. The OWA combination operator in Eq. (8) ignores the fact that most of the GIS-based decision-making problems require a set of different weights to be assigned to criterion maps layers. In order to extend the conventional OWA approach, it is necessary to fuse the ‘criterion weights’ (importances), W, into the OWA procedure. In this regard a criterion weight modification approach for the inclusion of criterion weights into the OWA operator is as follows:

9 $${\mathrm{V}}_{\mathrm{j }}=\mathrm{Q }\left(\genfrac{}{}{0pt}{}{{\sum }_{\mathrm{I}=1}^{\mathrm{i}}{\mathrm{u}}_{\mathrm{I}}}{\overline{{\sum }_{\mathrm{I}=1}^{\mathrm{n}}{\mathrm{u}}_{\mathrm{I}}}}\right)-\mathrm{Q }\left(\genfrac{}{}{0pt}{}{{\sum }_{\mathrm{I}=1}^{\mathrm{j}-1}{\mathrm{u}}_{\mathrm{I}}}{\overline{{\sum }_{\mathrm{I}=1}^{\mathrm{n}}{\mathrm{u}}_{\mathrm{I}}}}\right)$$

where ${\mathrm{u}}_{\mathrm{j}}$ is the reordered jth criterion weight, ${\mathrm{w}}_{\mathrm{j}}$, according to the reordered ${\mathrm{z}}_{\mathrm{ij}}.$ Considering ${\mathrm{Q}(\mathrm{p})=\mathrm{p}}^{\mathrm{x}}$ for x > 0, Eq. (2) can be simplified to:

10 $${{\mathrm{V}}_{\mathrm{j }}=\left(\genfrac{}{}{0pt}{}{{\sum }_{\mathrm{I}=1}^{\mathrm{i}}{\mathrm{u}}_{\mathrm{I}}}{\overline{{\sum }_{\mathrm{I}=1}^{\mathrm{n}}{\mathrm{u}}_{\mathrm{I}}}}\right)}^{\mathrm{x}}- {\left(\genfrac{}{}{0pt}{}{{\sum }_{\mathrm{I}=1}^{\mathrm{j}-1}{\mathrm{u}}_{\mathrm{I}}}{\overline{{\sum }_{\mathrm{I}=1}^{\mathrm{n}}{\mathrm{u}}_{\mathrm{I}}}}\right)}^{\mathrm{x}}$$

Accordingly, given the sets of criterion weights, W, and order weights, $\mathrm{V}$, the OWA operator can be defined as:

11 $${\mathrm{OWA}}_{\mathrm{i}} \sum_{\mathrm{j}=1}^{\mathrm{n}} =\left({\left(\genfrac{}{}{0pt}{}{{\sum }_{\mathrm{I}=1}^{\mathrm{i}}{\mathrm{u}}_{\mathrm{I}}}{\overline{{\sum }_{\mathrm{I}=1}^{\mathrm{n}}{\mathrm{u}}_{\mathrm{I}}}}\right)}^{\mathrm{x}}- {\left(\genfrac{}{}{0pt}{}{{\sum }_{\mathrm{I}=1}^{\mathrm{j}-1}{\mathrm{u}}_{\mathrm{I}}}{\overline{{\sum }_{\mathrm{I}=1}^{\mathrm{n}}{\mathrm{u}}_{\mathrm{I}}}}\right)}^{\mathrm{x}}\right){\mathrm{z}}_{\mathrm{ij}}$$

OWA provides a tool for generating a wide range of decision strategies in a decision strategy space, by applying a set of order weights to criteria that are ranked in ascending order on a pixel-by-pixel basis. Using the fuzzy decision rules for spatial aggregation provides further flexibility for the aggregation technique and improves the method's functionality^72^. We refer to Feizizadeh and Kienberger^73^, for detailed information and the mathematical background of the OWA method. In order to apply the OWA as a spatial aggregation method, the standardized criteria were aggregated based on the computed FANP weights.

### Ethics approval and consent to participate (human ethics, animal ethics or plant ethics)

The study protocol conformed to the ethical guidelines of the 1975 Declaration of Helsinki, and the Ethics Committee of Shahid Beheshti University of Medical Sciences under the ethics code IR.SBMU.RETECH.REC.1394.121 approved it. Researches considered all the principles of medical ethics, including the consent of the participants, the confidentiality of personal data and free measurements.

### Statement on the experimental human participant

We state that the use of experimental human participants is not relevant to this study. We employed a secondary dataset obtained from a health survey of the national STEPS project which is carried out by the Iranian Ministry of Health and Medical Education based on the international standards to reserve all human rights of the participants.

## Results

This research spatially relates several significant environmental aspects, particularly saline flow sources and salt-dust scatter sources, to their respective impacts on human health in Shabestar County. Although our results have demonstrated a decline in changes to the lake level, the areas of salt lands are still increasing significantly. Figure 6 shows the extent of salt-dust scatter sources in the north area of the lake from 2012 to 2020. The water level changes and exposition of salat lands has also represented in the Fig. 2 in the section of case study statement. The detailed results showed that the water body of the lake changed several times over the studied period based on the annual precipitation. Table 5 also shows the computed environmental changes and the number of patients in settlements around the lake over the years. As Table 5 shows, the exposed salt-dust scatter hotspots increased significantly over the past decade. Based on Table 5, the area of salt-dust scatter sources in 2012 was computed to be 103.24 km^2^, which increased to 229, 345, and 343.6 km^2^ in 2016, 2017, and 2018, respectively. The salt-dust scatter sources reduced in 2019 and 2020 to 189.70 and 111.51 km^2^, respectively (Fig. 2).

**Table 5:** Time-series analysis for salt-dust releases in the area north of the lake, as represented in Fig. 6, and percentage of hypertensive patients in each year obtained by the STEPS survey and health centers, as represented in Fig. 7.

Year	Salt-land area (km^2^)	Water-body area(km^2^)	hypertensive female patients	hypertensive male patients	Sum hypertensive patients	Major age group of hypertensive patients
2012	103.24	434.56	142	108	250	35–64
2013	135.14	603.33	225	156	381	35–64
2014	185	406.40	75	40	115	35–64
2015	178	389.25	560	375	935	35–64
2016	229	330.14	784	497	1281	35–64
2017	345	280.56	1136	1017	2153	35–64
2018	343.6	260.3	1076	1010	2086	35–64
2019	189.70	540.9	1225	1115	2340	35–64
2020	111.51	654.85	1155	770	1925	35–64

**Figure 7:**
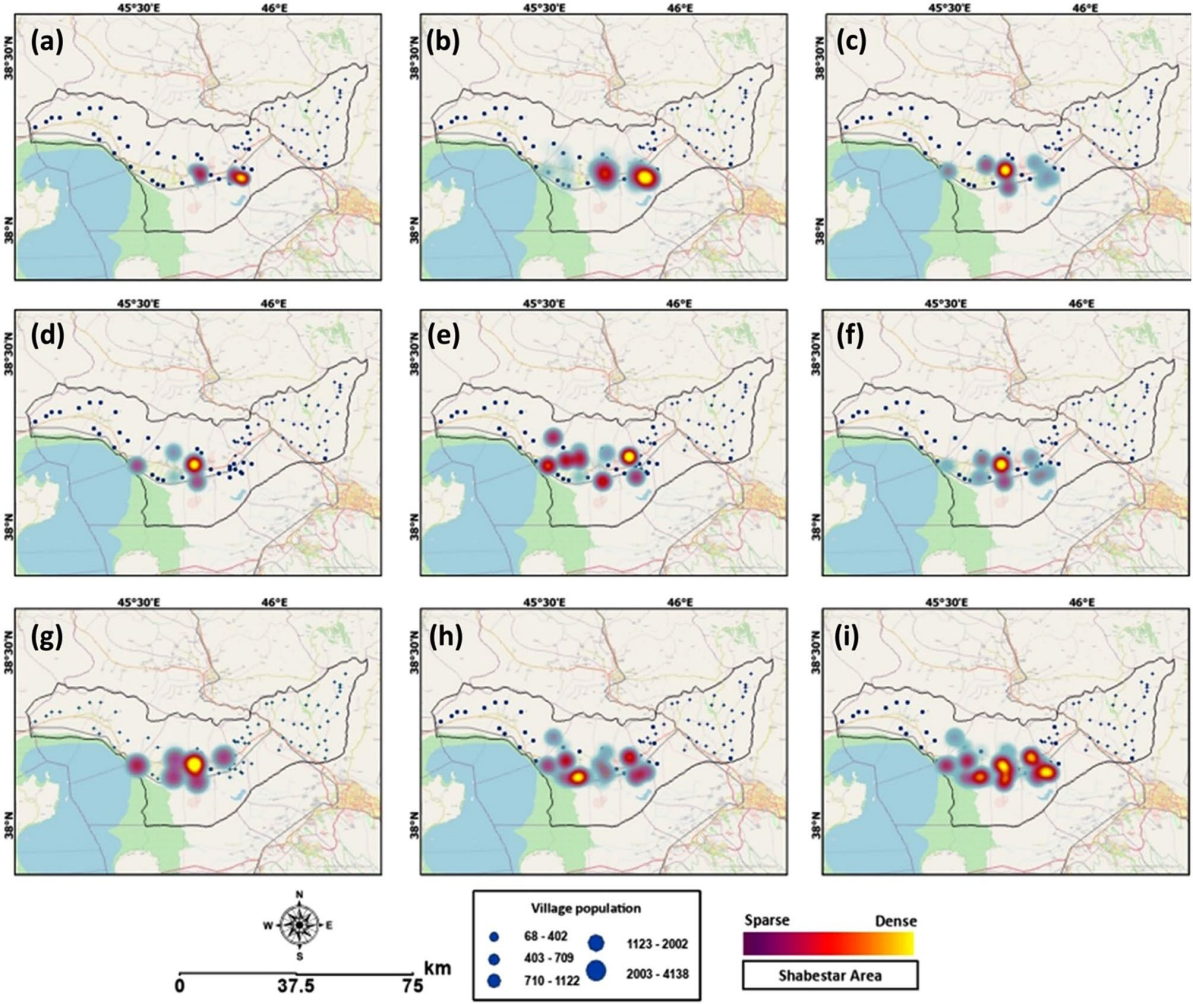
Results of the time-series analyzing hypertension in the study area in: (**a**) 2012, (**b**) 2013, (**c**) 2014, (**d**) 2015, (**e**) 2016, (**f**) 2017, (**g**) 2018, (**h**) 2019, and (**i**) 2020. These maps were developed based on the results obtained from the STEPS survey and health care centers for monitoring the impacts of the Lake Urmia drought on human health in Shabestar County. The map is created in ESRI- Arc GIS version 10.7 under licenses of Humboldt-Universität zu Berlin (https://desktop.arcgis.com/).

Figure 7 also represents the trend of hypertensive patients from 2012 to 2020. As can be seen from this figure, the expansion of the salt-dust scatters has impacted the residents’ health, as confirmed by the increasing number of hypertensive patients. As indicated in Table 5, the trend of hypertensive patients in the 12000 STEPS participants shows that in the year 2012, only 250 patients (2.09%) suffered from hypertension. This number reached 1925 (16.05%) in 2020. Our results also showed 2340 hypertensive patients (19.5%) in 2019, 2086 patients (17.39%) in 2018, and 2153 (17.95%) patients in 2017, which shows that higher numbers of hypertensive patients coincide with times when the lake was facing a very critical condition, and the expansion of salt lands was profound.

**Figure 8:**
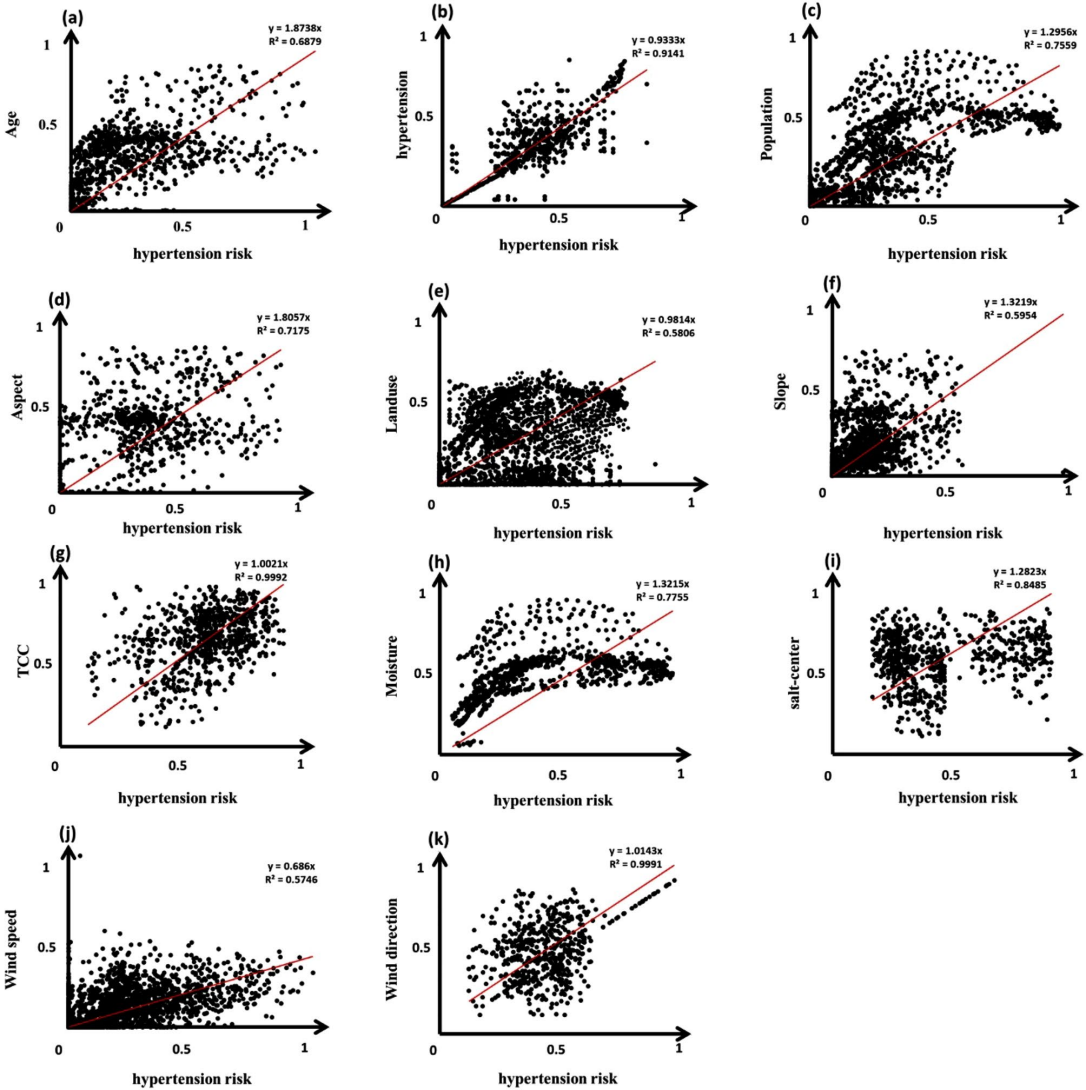
Results of the spatial correlation of the aggregated hypertension map with the selected criteria for health vulnerability and risk mapping: (**a**) age group, (**b**) current status of hypertension, (**c**) population density, (**d**) slope-aspect, (**e**) land use/cover, (**f**) slope, (**g**) vegetation continuous fields, (**h**) soil moisture, (**i**) distance to salt-centers, (**j**) wind speed, and (**k**) wind direction. These plots indicate the contribution of each criterion to the vulnerability and health risk. As can be observed from the plots, all criteria positively correlate with average hypertension.

Figure 8 shows the computed spatial correlation between the average hypertension level and the selected vulnerability and health risk mapping (VHRM) criteria. The results demonstrate the relationship between the lake drying and the prevalence of hypertension in the surrounding areas. There is a significant relationship between decreasing lake levels and increasing percentages of patients with hypertension. As indicated in Figs. 7 and 8j,k, the wind speed and direction have a substantial spatial correlation with the number of hypertensive patients. According to these maps, the wind speed and distance from salt/dust scatter sources clearly indicate the significance of these sources on the health of the nearby residents. Furthermore, ancillary information has shown that the number of female patients with hypertension is higher than the number of affected men, which indicates an increased sensitivity of females to these environmental challenges. It must be noted that all hypertension patients, both male and female, were aged between 35 and 70.

**Figure 9:**
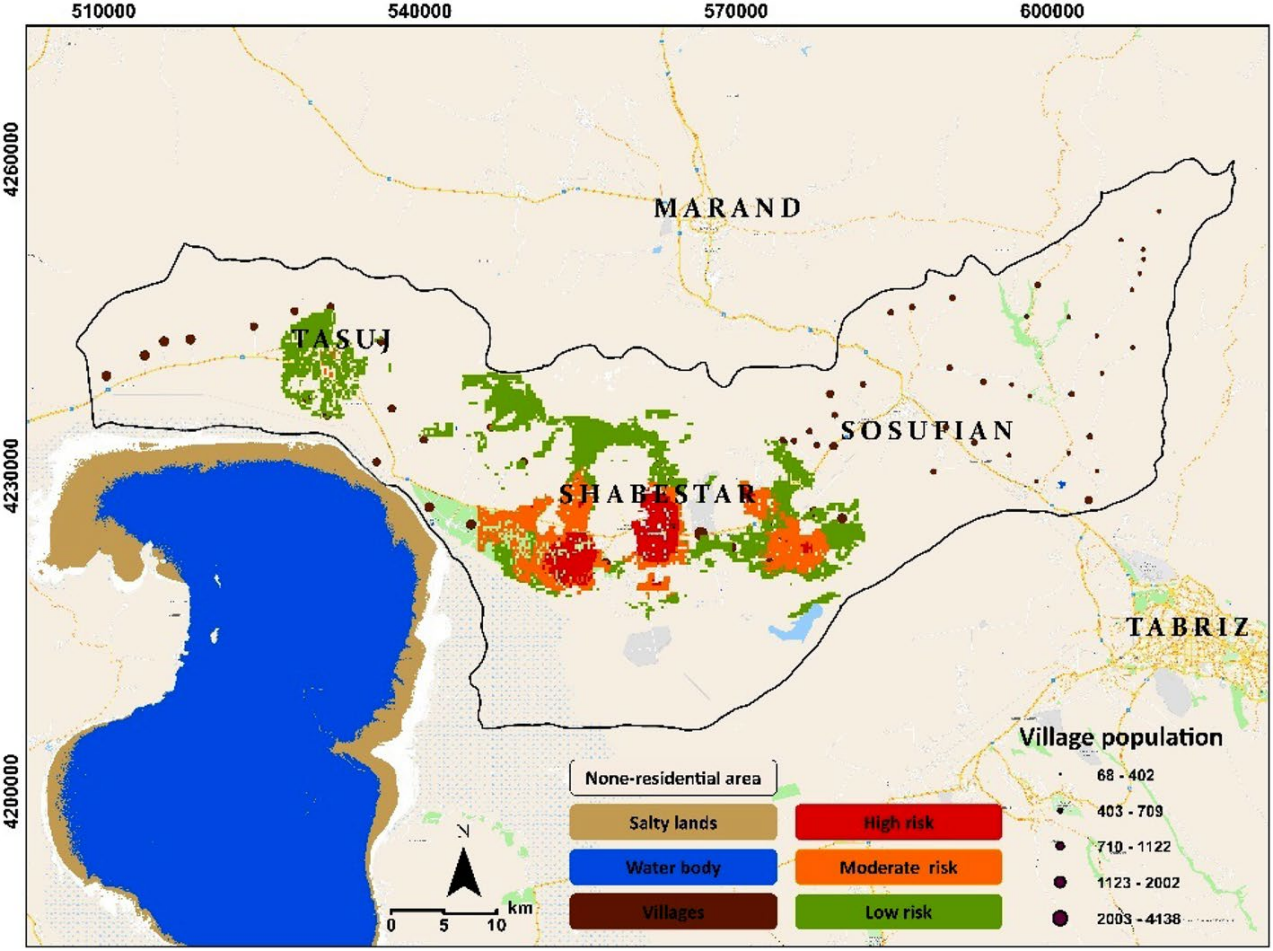
Results of GIS-MCDA-based vulnerability and health risk mapping based on the aggregation of the affecting environmental criteria as represented in Fig. 5. The map shows areas that face vulnerability and health risks due to salt-dust distribution. The map is created in ESRI- Arc GIS version 10.7 under licenses of Humboldt-Universität zu Berlin (https://desktop.arcgis.com/).

Figure 9 shows the result of the GIS-MCDA-based vulnerability and health risk mapping. According to this map, the cities of Shabestar, Vaigahn, Shandabad, and Sis lie within areas with a high risk of salt-dust scatters. These cities are located in the northern area of the Lake Urmia and are thus impacted by the local climate condition called the “Sea breeze”, which is a local wind from lake to land and one of the most critical causal climate characteristics leading to the dispersion of salt-dust. The map shown in Fig. 9 also shows the effect of salt-dust scatters on the public health in the study area. Based on this figure and the results of trend assessments, it can be expected that the area will face a serious threat to its residents’ health in the coming years that will require the careful attention of authorities and decision-makers. According to Fig. 9, in deposit of the distance from lake, there are several villages in the eastern area that are also identified as highly vulnerable area for the salt/dust scattering. Our investigation and field evaluation shows that the significance of population in age groups older than 60 years in these villages for identifying them as highly vulnerability and health risks due to salt-dust distribution. Our field investigation and interview with local residents indicated that due intensive impacts of lake drought on agricultural activities over the past decades, many young generation immigrated to the Tabriz, Tehran and other large cities in search of job and a better life quality. This socioeconomic issue turned to regular process in village areas in the vicinity of Lake Urmia over the past years which also resulted serious challenge for the host cities such as Tabriz and Urmia. Such an intensive immigrant resulted developing of the enormous informal settlements around the Tabriz which accordingly costed significant impacts for the sustainable urban development in the city. Based on this situation there is unbalanced development in this city which resulted different lifestyle and life quality in Tabriz^46^.

## Discussion

The results indicated a significant relationship between decreasing lake water levels and increasing hypertension, as depicted in Figs. 6, 7 and Table 5. We also identified a positive correlation between the decrease in the surface area of Lake Urmia and an increase in the number of patients. Results of spatial correlation analysis of the average hypertension (Fig. 8) indicated that there is liner correlation between average hypertension and current status of hypertension, age of local population, slope as well as wind speed and direction, which clearly indicate the significance of physical and environmental characteristics the study area and number of hypertension patients. The obtained mapping results show the number of hypertensive patients in different areas within the study area, whereby the areas are classified from diffuse to dense based on the number of patients. The age map indicates where people are at risk of developing hypertension based on their age, ranging from low to severe. According to this classification, people aged between 35 and 50 have a low to moderate risk and people aged 50 to 70 have a moderate to high risk of developing respiratory diseases and hypertension. In addition, the demographic analysis showed that people between the ages of 35 and 70 were most likely to have hypertension. Furthermore, throughout the entire study period, the number of female residents with hypertension outweighed the number of men suffering from hypertension. Our study also ascertained that the percentage of residents with hypertension, proximity to salt diffusion centers, wind direction, and age were the most critical factors determining hypertension risk in the area. Several studies have recently been conducted on the relationship between spatial dimensions and patient prevalence^67,74–78^.

Review of research laterite regarding the air pollution resulted from the saline flow resources of the lake Urmia indicated the significant increase in aerosol pollution around the lake over the last 10 years. The significance concern of harvesting deposited salts from the exposed saline flows from the lake bed. Based on these studies, the dust emission phenomena could become more severe and widespread because of uncovering and drying of sludge existing under the salt layers^79–87^. According to Gholampour et al.^79^, in the Tabriz city and suburbs areas of the Lake Urmia, water soluble ions accounted to be approximately 20% ± 10% of total TSP mass and 25% ± 12% of total PM_10_ mass. They also evaluated the characterization of saline dust emission resulted from Urmia Lake drying analysis. They aimed to analysis particulate matter (PM) in the vicinity of lake Urmia for the year of 2013. According to their study, the highest concentration of PM was observed during the summer season (521.6, 329.1, 42.6, and 36.5 for TSP, PM_10_, PM_2.5_, and PM_1_, respectively)^79–81^. According to Alizade Govarchin Ghale et al.^84,85^, there was an inverse relationship between water level fluctuations of the lake and aerosol pollution. They also observed that the mean of aerosol optical depth increased to 0.42 from the period of 2010 to 2019. Based on this study, the annual mean PM_10_ concentration has increased after 2013 significantly. They pointed out the maximum 24-h mean PM_10_ concentration to be in December of 2015 to be about 876.13 μg/m^3^^84,85^. As our study as well as the related studies also indicated, the water level of Lake Urmia dramatically dropped from 2013 to 2015 which accordingly exposed incisive saline flow sources.

From the methodological perspective, results of this study pointed out that remote sensing enables us to represent the spatial and temporal relationships of various phenomena, which is critical for monitoring the environmental impacts on human health. Our results indicated that the integrated GIS and remote sensing (or Geoinformation) approach could be applied for human health monitoring and vulnerability and risk mapping through forecasting the future environmental conditions and their impacts. Healthcare planning and GIS are two related fields that both depend on spatial data. Spatial features in healthcare include the geographical location of the patient and the distribution of healthcare facilities, the management of which is supported by various GIS functions and models. One of the issues that justifies the existence of an appropriate GIS system in the healthcare field, is less reliable population data, sensitivity to lost data, and data elimination in sparsely populated areas^78^. The field of epidemiology makes use of the capabilities of GIS, which has been one of the most significant investigation domains in the past two decades^88^. Considering that epidemiology is completely related to the spatial dimension, questions about the origin and prevalence patterns of diseases can thus be answered based on a GIS spatial analysis^47^. Access to accurate data can help identify and treat disease and prevent the occurrence and spread of disease using screening and prevention measures. In this regard, strong statistical approaches were developed to identify spatial or spatiotemporal clusters^88^.

The combination of public health and informatics shows how numerous health problems affect certain populations and identifies the trends of this impact^24^. Planning the allocation of healthcare resources is critical to assure maximum equality in healthcare access^75,90^. GIS has played a crucial role in managing various health issues (e.g., the impact of COVID-19 on human health, as a recent example), which has led researchers to consider the spatial relationships between health and various influencing factors affecting it. In this context, GIS-based analysis plays a principal role in the spatial modeling of pandemics to support epidemic prevention and control measures, determine the spatial allocation of health resources and recognize social behavior patterns^89^. GIS has played a significant role in analyzing health issues by applying analytical approaches and evaluating the spatial inequality of access to healthcare facilities. The use of GIS for surveying healthcare planning problems is vital to relevant studies^65^. It is vital that all patients are able to access healthcare facilities and political policy should ensure that access to adequate healthcare is available to all. In this regard, to define a unique theoretical geographic unit for the healthcare market (e.g., primary care service areas, hospital service areas, and cancer service areas), it is necessary for researchers and policy-makers to make use of a GIS spatial analysis.

Our study faces some limitations in terms of data availability. The health data we have is of good quality (reference), but it only represents a small sample of the total population. Not all age groups are covered, for example. There could also be a temporal lag between exposure to salt dust, or other variables that affect health, and suffering the negative health effects of these impacts. Long-term analyses should be established to address this effect in more detail. Moreover, we had to approximate the determination factors of hypertension using indicators because of missing modeling results on salt dust distribution patterns, e.g., wind speed, land use. A salt-dust diffusion and impact model is a necessary next step to better understand the actual spatial and temporal effects of salt dust. Finally, in several steps of the analysis, expert knowledge was used to inform weight adjustments, our choice of indicators, and to define parameters. We took care to do this with transparency towards the experts and leveraging the current body of knowledge. Monitoring future health outcomes in relation to the proposed risk map and transferring the presented approach to other regions may be useful to further develop the approach.

## Conclusion

The main objective of this research was to monitor the spatiotemporal impacts of the Lake Urmia drought on the human health of the local population in Shabestar County. As indicated in the research methodology, we also carried out GIS-MCDA-based vulnerability and health risk mapping (VHRM) to ascertain the future health risks in the study area. We compared the resulting average hypertensive map against the selected criteria to analyze the spatial correlation of the number of hypertensive patients in the study area with selected criteria for VHRM. According to our results, the main conclusion to be drawn from this study was the direct association between Lake Urmia drought, the expansion of salt diffusion centers and their respective salt-dust scatters, and the increase in hypertension risk in Shabestar County. The study indicated that the appearance of salt diffusion centers around the lake, coupled with certain environmental criteria (e.g., wind direction and geomorphological characteristics), can significantly contribute to the increase in hypertension in all area of Lake Urmia basin which lead us to conclude that the lake drought will further impact the health of the residents around the lake in years to come. It must be highlighted that about 7.3 million people live around Lake Urmia, and we only evaluated a small fraction of Shabestar County based on the data available through the health care centers and STEPS survey. Thus, we expect the actual number of hypertension patients to be much higher than the number of patients identified in this study, although the percentages certainly indicate a general trend. Therefore, the results of this study are critical for decision-makers and authorities because they provide detailed information regarding the environmental impacts of the lake drought on salt-dust-scatter and its respective impacts on the health of the residents in Shabestar County.

Even though the results show a significant correlation between the lake drought and increasing numbers of hypertension patients in Shabestar County, it must be said that hypertension can also be impacted by lifestyle choices, nutrition, and socioeconomic status. It might even be worthwhile considering the impacts of the current economic situation in Iran that led to a drop-in revenue, a depreciation of the national currency, an increase in inflation, and unemployment. These issues significantly impact the quality of life by reducing people's overall wellbeing and their capacity to acquire basic necessities such as nutritious food, healthcare, and medicine^90^. Therefore, we conclude that a comprehensive study that considers lifestyle and nutrition might be required to provide further details to thoroughly analyze public health in this area. The results of this initial research could already be used in planning measures to allocate services to areas with a high risk of hypertension to identify and mitigate possible damage caused by consequences of the lake drought through strategies such as preventing the destruction of halophyte plants around the lake. This study clearly indicates the significant impacts of environmental issues associated with dying lakes on the health of the residents. In considering the extensive impacts of global climate change and the issues associated with dying lakes, we conclude that the proposed integrated Geoinformation approach can be efficiently applied to investigate the relationship between environmental degradation and scenario-based human health vulnerability and risk mapping in other areas with dying lakes around the world.

## Data Availability

The GIS format of the environmental data will be available by request to the corresponding author. Request for the health survey dataset must be sent to the Iranian Ministry of Health and Medical Education as data holder and authority. Data will be available on reasonable request, research proposals from universities /institutes or individual researchers through the following link: https://en.tums.ac.ir/.
